# (*E*)-*N*′-(2-Chloro­benzyl­idene)-3,5-di­hydroxy­benzohydrazide dihydrate

**DOI:** 10.1107/S1600536811042681

**Published:** 2011-10-22

**Authors:** Ling Yuan, Yi Nan, Jing-Yuan Li, Xiu-Lan Huang

**Affiliations:** a Pharmacy College of Ningxia Medical University, Yinchuan, Ningxia Province 750004, People’s Republic of China; b Traditional Chinese Medicine College of Ningxia Medical University, Ningxia Province, 750004, People’s Republic of China; c Minority Traditional Medical Center of Minzu University of China, Beijing 100081, People’s Republic of China

## Abstract

In the Schiff base mol­ecule of the title compound, C_14_H_11_ClN_2_O_3_·2H_2_O, the benzene rings form a dihedral angle of 20.6 (1)°. The water molecules of crystallization are involved in the formation of a three-dimensional hydrogen-bonding network *via* O—H⋯O and N—H⋯O hydrogen bonds.

## Related literature

For general background to Schiff base compounds, see: Brückner *et al.* (2000 ▶); Harrop *et al.*(2003 ▶); Zhang *et al.* (2008 ▶). For related structures, see: Diao *et al.* (2007 ▶); Jiang *et al.* (2008 ▶); Huang *et al.* (2008 ▶); Deng *et al.* (2009 ▶).
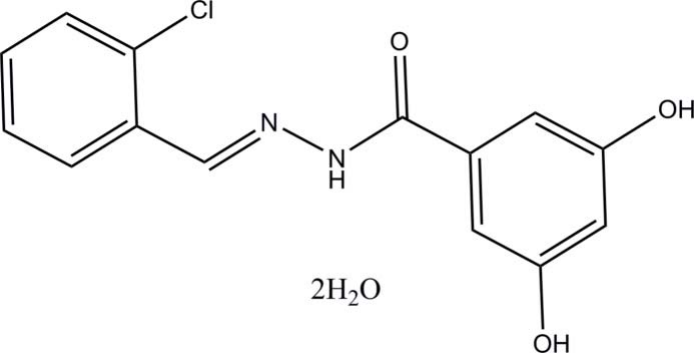


## Experimental

### 

#### Crystal data

C_14_H_11_ClN_2_O_3_·2H_2_O
*M* *_r_* = 326.73
Monoclinic, 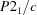

*a* = 8.023 (2) Å
*b* = 11.852 (4) Å
*c* = 16.318 (5) Å
β = 100.387 (4)°
*V* = 1526.1 (8) Å^3^
*Z* = 4
Mo *K*α radiation
μ = 0.28 mm^−1^
*T* = 296 K
0.44 × 0.12 × 0.07 mm

#### Data collection

Bruker APEXII CCD diffractometer
12729 measured reflections
3522 independent reflections
2286 reflections with *I* > 2σ(*I*)
*R*_int_ = 0.040

#### Refinement

*R*[*F* ^2^ > 2σ(*F* ^2^)] = 0.051
*wR*(*F* ^2^) = 0.140
*S* = 1.03
3522 reflections
199 parameters
H-atom parameters constrained
Δρ_max_ = 0.32 e Å^−3^
Δρ_min_ = −0.33 e Å^−3^

### 

Data collection: *APEX2* (Bruker, 2005 ▶); cell refinement: *APEX2*; data reduction: *SAINT* (Bruker, 2005 ▶); program(s) used to solve structure: *SHELXS97* (Sheldrick, 2008 ▶); program(s) used to refine structure: *SHELXL97* (Sheldrick, 2008 ▶); molecular graphics: *SHELXTL* (Sheldrick, 2008 ▶); software used to prepare material for publication: *SHELXL97*.

## Supplementary Material

Crystal structure: contains datablock(s) I, global. DOI: 10.1107/S1600536811042681/cv5172sup1.cif

Structure factors: contains datablock(s) I. DOI: 10.1107/S1600536811042681/cv5172Isup2.hkl

Supplementary material file. DOI: 10.1107/S1600536811042681/cv5172Isup3.cml

Additional supplementary materials:  crystallographic information; 3D view; checkCIF report

## Figures and Tables

**Table 1:** Hydrogen-bond geometry (Å, °)

*D*—H⋯*A*	*D*—H	H⋯*A*	*D*⋯*A*	*D*—H⋯*A*
O2—H2*B*⋯O5	0.82	1.87	2.674 (3)	168
O3—H3*A*⋯O1^i^	0.82	1.85	2.665 (2)	169
N2—H2*A*⋯O2^ii^	0.86	2.36	3.196 (3)	164
O4—H4*B*⋯O1	0.85	2.12	2.960 (4)	171
O5—H5*A*⋯O4^iii^	0.85	1.99	2.816 (4)	163
O5—H5*B*⋯O3^iv^	0.85	2.14	2.902 (3)	150

Symmetry codes: (i) 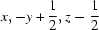; (ii) 
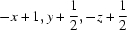
; (iii) 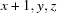; (iv) 
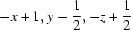
.

**Fig. 1.**
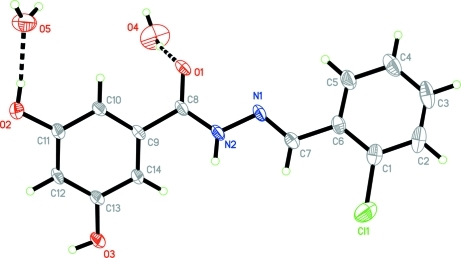
The molecular structure of (I) showing the atomic numbering and 30% probability displacement ellipsoids.

## supplementary crystallographic information

### Comment

Schiff base compounds are known to exhibit antibacterial and antitumor properties (Brückner *et al.*, 2000; Harrop *et al.*, 2003; Zhang *et al.*, 2008). In order to expand this filed, we report here the structure of the title compound (I).

In (I) (Fig. 1), all bond lengths and angles are normal and comparable with those found in the related compounds (Diao *et al.*, 2007; Deng *et al.*, 2009; Huang *et al.*, 2008, Jiang *et al.*, 2008). In the Shiff base molecule, two benzene rings form a dihedral angle of 20.6 (1)°.

In the crystal structure, intermolecular O—H···O and N—H···O hydrogen bonds (Table 1) consolidate the crystal packing.

### Experimental

2-Chlorobenzaldehyde (0.1 mmol, 14.1 mg) and 3,5-dihydroxybenzhydrazide (0.1 mmol, 16.8 mg) were dissolved in a methanol solution (10 ml). The mixture was stirred at room temperature for 1 h and filtered. After keeping the filtrate in air for three days, yellow crystals were formed.

### Refinement

H atoms were placed in calculated positions (C—H 0.93 Å; N—H 0.86 Å; O—H 0.82 Å) and were included in the refinement in the riding model approximation, with *U*_iso_(H)=) =1.2-1.5*U*_eq_ of the parent atom.

### Crystal data

**Table:**

C_14_H_11_ClN_2_O_3_·2H_2_O	*F*(000) = 680
*M**_r_* = 326.73	*D*_x_ = 1.422 Mg m^−^^3^
Monoclinic, *P*2_1_/*c*	Mo *K*α radiation, λ = 0.71073 Å
Hall symbol: -P2ybc	Cell parameters from 4061 reflections
*a* = 8.023 (2) Å	θ = 2.5–27.1°
*b* = 11.852 (4) Å	µ = 0.28 mm^−^^1^
*c* = 16.318 (5) Å	*T* = 296 K
β = 100.387 (4)°	Stick, yellow
*V* = 1526.1 (8) Å^3^	0.44 × 0.12 × 0.07 mm
*Z* = 4	

### Data collection

**Table:**

Bruker APEXII CCD diffractometer	2286 reflections with *I* > 2σ(*I*)
Radiation source: fine-focus sealed tube	*R*_int_ = 0.040
graphite	θ_max_ = 27.6°, θ_min_ = 2.1°
φ and ω scans	*h* = −10→10
12729 measured reflections	*k* = −15→15
3522 independent reflections	*l* = −20→21

### Refinement

**Table:**

Refinement on *F*^2^	Primary atom site location: structure-invariant direct methods
Least-squares matrix: full	Secondary atom site location: difference Fourier map
*R*[*F*^2^ > 2σ(*F*^2^)] = 0.051	Hydrogen site location: inferred from neighbouring sites
*wR*(*F*^2^) = 0.140	H-atom parameters constrained
*S* = 1.03	*w* = 1/[σ^2^(*F*_o_^2^) + (0.044*P*)^2^ + 1.2*P*] where *P* = (*F*_o_^2^ + 2*F*_c_^2^)/3
3522 reflections	(Δ/σ)_max_ < 0.001
199 parameters	Δρ_max_ = 0.32 e Å^−^^3^
0 restraints	Δρ_min_ = −0.33 e Å^−^^3^

### Special details

**Table:**

Geometry. All e.s.d.'s (except the e.s.d. in the dihedral angle between two l.s. planes) are estimated using the full covariance matrix. The cell e.s.d.'s are taken into account individually in the estimation of e.s.d.'s in distances, angles and torsion angles; correlations between e.s.d.'s in cell parameters are only used when they are defined by crystal symmetry. An approximate (isotropic) treatment of cell e.s.d.'s is used for estimating e.s.d.'s involving l.s. planes.
Refinement. Refinement of *F*^2^ against ALL reflections. The weighted *R*-factor *wR* and goodness of fit *S* are based on *F*^2^, conventional *R*-factors *R* are based on *F*, with *F* set to zero for negative *F*^2^. The threshold expression of *F*^2^ > σ(*F*^2^) is used only for calculating *R*-factors(gt) *etc*. and is not relevant to the choice of reflections for refinement. *R*-factors based on *F*^2^ are statistically about twice as large as those based on *F*, and *R*- factors based on ALL data will be even larger.

### Fractional atomic coordinates and isotropic or equivalent isotropic displacement parameters (Å^2^)

**Table:**

	*x*	*y*	*z*	*U*_iso_*/*U*_eq_	
Cl1	0.07549 (13)	0.73732 (7)	0.46696 (6)	0.0836 (3)	
O1	0.4482 (3)	0.20583 (16)	0.45642 (10)	0.0555 (5)	
O2	0.7564 (2)	0.07180 (15)	0.22642 (10)	0.0508 (5)	
H2B	0.7932	0.0426	0.2716	0.076*	
O3	0.3480 (3)	0.33745 (18)	0.10033 (10)	0.0615 (6)	
H3A	0.3864	0.3177	0.0593	0.092*	
N1	0.3161 (2)	0.41028 (17)	0.48357 (10)	0.0374 (4)	
N2	0.3589 (2)	0.37857 (17)	0.40850 (10)	0.0368 (4)	
H2A	0.3450	0.4252	0.3674	0.044*	
C1	0.1081 (3)	0.6554 (2)	0.55602 (17)	0.0472 (6)	
C2	0.0554 (4)	0.6968 (3)	0.6271 (2)	0.0621 (8)	
H2	0.0022	0.7666	0.6257	0.074*	
C3	0.0818 (4)	0.6351 (3)	0.6986 (2)	0.0678 (9)	
H3	0.0483	0.6635	0.7462	0.081*	
C4	0.1571 (4)	0.5318 (3)	0.70062 (18)	0.0687 (9)	
H4	0.1738	0.4897	0.7495	0.082*	
C5	0.2090 (4)	0.4891 (3)	0.63044 (16)	0.0550 (7)	
H5	0.2596	0.4184	0.6326	0.066*	
C6	0.1866 (3)	0.5505 (2)	0.55659 (14)	0.0382 (5)	
C7	0.2396 (3)	0.5047 (2)	0.48164 (14)	0.0371 (5)	
H7	0.2171	0.5453	0.4321	0.044*	
C8	0.4220 (3)	0.2757 (2)	0.39941 (12)	0.0353 (5)	
C9	0.4635 (3)	0.25014 (19)	0.31552 (12)	0.0326 (5)	
C10	0.5859 (3)	0.16929 (19)	0.31167 (12)	0.0347 (5)	
H10	0.6357	0.1302	0.3592	0.042*	
C11	0.6334 (3)	0.14750 (19)	0.23525 (13)	0.0351 (5)	
C12	0.5573 (3)	0.2046 (2)	0.16457 (12)	0.0387 (5)	
H12	0.5916	0.1910	0.1140	0.046*	
C13	0.4305 (3)	0.2818 (2)	0.16880 (12)	0.0387 (5)	
C14	0.3828 (3)	0.3059 (2)	0.24457 (13)	0.0393 (5)	
H14	0.2981	0.3585	0.2477	0.047*	
O4	0.2697 (4)	−0.0135 (3)	0.4316 (2)	0.1159 (11)	
H4A	0.3177	−0.0688	0.4595	0.174*	
H4B	0.3266	0.0473	0.4339	0.174*	
O5	0.9176 (3)	−0.00817 (18)	0.37266 (13)	0.0690 (6)	
H5A	1.0182	−0.0070	0.4001	0.104*	
H5B	0.8554	−0.0465	0.3993	0.104*	

### Atomic displacement parameters (Å^2^)

**Table:**

	*U*^11^	*U*^22^	*U*^33^	*U*^12^	*U*^13^	*U*^23^
Cl1	0.1051 (7)	0.0521 (5)	0.0969 (7)	0.0157 (5)	0.0274 (6)	0.0134 (4)
O1	0.0881 (14)	0.0572 (11)	0.0272 (8)	0.0154 (10)	0.0261 (9)	0.0084 (8)
O2	0.0624 (12)	0.0562 (11)	0.0360 (9)	0.0213 (9)	0.0146 (8)	−0.0016 (8)
O3	0.0820 (14)	0.0817 (14)	0.0224 (8)	0.0345 (12)	0.0139 (8)	0.0076 (8)
N1	0.0404 (11)	0.0505 (12)	0.0244 (8)	0.0010 (9)	0.0141 (8)	−0.0046 (8)
N2	0.0438 (11)	0.0479 (11)	0.0217 (8)	0.0006 (9)	0.0140 (8)	−0.0011 (8)
C1	0.0379 (14)	0.0438 (14)	0.0619 (16)	−0.0085 (11)	0.0146 (12)	−0.0153 (12)
C2	0.0455 (16)	0.0534 (17)	0.093 (2)	−0.0089 (13)	0.0284 (16)	−0.0352 (17)
C3	0.0585 (19)	0.087 (2)	0.067 (2)	−0.0154 (17)	0.0349 (16)	−0.0362 (18)
C4	0.071 (2)	0.097 (3)	0.0449 (15)	0.0004 (19)	0.0279 (14)	−0.0074 (16)
C5	0.0568 (17)	0.0714 (19)	0.0414 (14)	0.0092 (14)	0.0212 (12)	−0.0046 (13)
C6	0.0311 (12)	0.0477 (13)	0.0384 (12)	−0.0053 (10)	0.0131 (9)	−0.0095 (10)
C7	0.0366 (12)	0.0455 (13)	0.0311 (11)	−0.0054 (11)	0.0114 (9)	−0.0022 (9)
C8	0.0394 (12)	0.0451 (13)	0.0240 (10)	0.0002 (10)	0.0129 (9)	−0.0016 (9)
C9	0.0368 (12)	0.0419 (12)	0.0209 (9)	−0.0035 (10)	0.0103 (8)	−0.0017 (8)
C10	0.0403 (13)	0.0412 (12)	0.0235 (9)	0.0004 (10)	0.0081 (9)	0.0006 (9)
C11	0.0406 (13)	0.0376 (12)	0.0290 (10)	−0.0009 (10)	0.0112 (9)	−0.0055 (9)
C12	0.0497 (14)	0.0474 (13)	0.0220 (9)	0.0015 (11)	0.0146 (9)	−0.0062 (9)
C13	0.0464 (13)	0.0481 (13)	0.0222 (10)	0.0042 (11)	0.0076 (9)	0.0001 (9)
C14	0.0464 (14)	0.0480 (13)	0.0257 (10)	0.0100 (11)	0.0124 (9)	−0.0005 (9)
O4	0.099 (2)	0.098 (2)	0.138 (3)	−0.0265 (18)	−0.0111 (19)	0.0009 (19)
O5	0.0685 (14)	0.0749 (14)	0.0616 (12)	0.0020 (11)	0.0063 (10)	0.0216 (11)

### Geometric parameters (Å, °)

**Table:**

Cl1—C1	1.728 (3)	C5—C6	1.391 (4)
O1—C8	1.235 (3)	C5—H5	0.9300
O2—C11	1.361 (3)	C6—C7	1.469 (3)
O2—H2B	0.8200	C7—H7	0.9300
O3—C13	1.362 (3)	C8—C9	1.497 (3)
O3—H3A	0.8200	C9—C10	1.382 (3)
N1—C7	1.274 (3)	C9—C14	1.388 (3)
N1—N2	1.383 (2)	C10—C11	1.392 (3)
N2—C8	1.338 (3)	C10—H10	0.9300
N2—H2A	0.8600	C11—C12	1.381 (3)
C1—C6	1.392 (4)	C12—C13	1.379 (3)
C1—C2	1.393 (4)	C12—H12	0.9300
C2—C3	1.361 (5)	C13—C14	1.388 (3)
C2—H2	0.9300	C14—H14	0.9300
C3—C4	1.363 (5)	O4—H4A	0.8500
C3—H3	0.9300	O4—H4B	0.8500
C4—C5	1.383 (4)	O5—H5A	0.8500
C4—H4	0.9300	O5—H5B	0.8500
			
C11—O2—H2B	109.5	N1—C7—H7	119.5
C13—O3—H3A	109.5	C6—C7—H7	119.5
C7—N1—N2	114.36 (19)	O1—C8—N2	122.96 (18)
C8—N2—N1	120.34 (18)	O1—C8—C9	121.1 (2)
C8—N2—H2A	119.8	N2—C8—C9	115.90 (19)
N1—N2—H2A	119.8	C10—C9—C14	121.39 (18)
C6—C1—C2	120.9 (3)	C10—C9—C8	117.35 (19)
C6—C1—Cl1	120.46 (19)	C14—C9—C8	121.3 (2)
C2—C1—Cl1	118.6 (2)	C9—C10—C11	118.7 (2)
C3—C2—C1	120.1 (3)	C9—C10—H10	120.6
C3—C2—H2	120.0	C11—C10—H10	120.6
C1—C2—H2	120.0	O2—C11—C12	117.05 (18)
C2—C3—C4	120.2 (3)	O2—C11—C10	122.6 (2)
C2—C3—H3	119.9	C12—C11—C10	120.4 (2)
C4—C3—H3	119.9	C13—C12—C11	120.16 (18)
C3—C4—C5	120.4 (3)	C13—C12—H12	119.9
C3—C4—H4	119.8	C11—C12—H12	119.9
C5—C4—H4	119.8	O3—C13—C12	122.25 (18)
C4—C5—C6	121.0 (3)	O3—C13—C14	117.4 (2)
C4—C5—H5	119.5	C12—C13—C14	120.3 (2)
C6—C5—H5	119.5	C9—C14—C13	118.9 (2)
C5—C6—C1	117.4 (2)	C9—C14—H14	120.6
C5—C6—C7	121.1 (2)	C13—C14—H14	120.6
C1—C6—C7	121.5 (2)	H4A—O4—H4B	116.3
N1—C7—C6	120.9 (2)	H5A—O5—H5B	109.2
			
C7—N1—N2—C8	−172.3 (2)	O1—C8—C9—C10	−24.0 (3)
C6—C1—C2—C3	0.6 (4)	N2—C8—C9—C10	154.7 (2)
Cl1—C1—C2—C3	−178.9 (2)	O1—C8—C9—C14	156.3 (2)
C1—C2—C3—C4	−1.1 (5)	N2—C8—C9—C14	−25.0 (3)
C2—C3—C4—C5	0.6 (5)	C14—C9—C10—C11	2.8 (3)
C3—C4—C5—C6	0.4 (5)	C8—C9—C10—C11	−176.9 (2)
C4—C5—C6—C1	−0.9 (4)	C9—C10—C11—O2	178.0 (2)
C4—C5—C6—C7	−179.2 (3)	C9—C10—C11—C12	−1.0 (3)
C2—C1—C6—C5	0.4 (4)	O2—C11—C12—C13	179.4 (2)
Cl1—C1—C6—C5	179.9 (2)	C10—C11—C12—C13	−1.6 (4)
C2—C1—C6—C7	178.7 (2)	C11—C12—C13—O3	−177.8 (2)
Cl1—C1—C6—C7	−1.7 (3)	C11—C12—C13—C14	2.4 (4)
N2—N1—C7—C6	−179.62 (19)	C10—C9—C14—C13	−2.0 (4)
C5—C6—C7—N1	−3.9 (4)	C8—C9—C14—C13	177.7 (2)
C1—C6—C7—N1	177.8 (2)	O3—C13—C14—C9	179.6 (2)
N1—N2—C8—O1	−1.5 (4)	C12—C13—C14—C9	−0.6 (4)
N1—N2—C8—C9	179.82 (19)		

### Hydrogen-bond geometry (Å, °)

**Table:**

*D*—H···*A*	*D*—H	H···*A*	*D*···*A*	*D*—H···*A*
O2—H2B···O5	0.82	1.87	2.674 (3)	168.
O3—H3A···O1^i^	0.82	1.85	2.665 (2)	169.
N2—H2A···O2^ii^	0.86	2.36	3.196 (3)	164.
O4—H4B···O1	0.85	2.12	2.960 (4)	171.
O5—H5A···O4^iii^	0.85	1.99	2.816 (4)	163.
O5—H5B···O3^iv^	0.85	2.14	2.902 (3)	150.

Symmetry codes: (i) *x*, −*y*+1/2, *z*−1/2; (ii) −*x*+1, *y*+1/2, −*z*+1/2; (iii) *x*+1, *y*, *z*; (iv) −*x*+1, *y*−1/2, −*z*+1/2.
